# Chronic Low-Dose Alcohol Consumption Attenuates Post-Ischemic Inflammation via PPARγ in Mice

**DOI:** 10.3390/ijms22105121

**Published:** 2021-05-12

**Authors:** Chun Li, Jiyu Li, Ethyn G. Loreno, Sumitra Miriyala, Manikandan Panchatcharam, Xiaohong Lu, Hong Sun

**Affiliations:** 1 Department of Cellular Biology and Anatomy, LSUHSC-Shreveport, Shreveport, LA 71130, USA; cli2@lsuhsc.edu (C.L.); jli6@lsuhsc.edu (J.L.); eloren@lsuhsc.edu (E.G.L.); smiriy@lsuhsc.edu (S.M.); mpanch@lsuhsc.edu (M.P.); 2 Department of Pharmacology, Toxicology & Neuroscience, Louisiana State University Health Sciences Center-Shreveport, Shreveport, LA 71130, USA; xlu1@lsuhsc.edu

**Keywords:** alcohol, ischemic stroke, PPARγ, inflammation

## Abstract

Ischemic stroke is one of the leading causes of death and permanent disability in adults. Recently, we found that light alcohol consumption (LAC) suppresses post-ischemic inflammatory response, which plays an important role in ischemic brain damage. Our goal was to determine the role of peroxisome proliferator-activated receptor-gamma (PPARγ) in the anti-inflammatory effect of LAC against transient focal cerebral ischemia. In in vivo study, male C57BL/6J wild type (WT) and endothelial-specific conditional PPARγ knockout mice were gavage fed with 0.7 g/kg/day ethanol or volume-matched water daily for 8 weeks. From the 7th week, 3 mg/kg/day GW9662 (a selective PPARγ antagonist) was intraperitoneally given for two weeks. Cerebral ischemia/reperfusion (I/R) injury and expression of manganese superoxide dismutase (MnSOD) and adhesion molecules, neutrophil infiltration, and microglial activation in the cerebral cortex before and following a 90 min unilateral middle cerebral artery occlusion (MCAO)/24 h reperfusion were evaluated. In in vitro study, the impact of chronic alcohol exposure on expression of PPARγ and MnSOD in C57BL/6J mouse brain microvascular endothelial cells (MBMVECs) was measured. PPARγ and MnSOD were significantly upregulated in the cerebral cortex of ethanol-fed WT mice and low-concentration ethanol-exposed C57BL/6J MBMVECs. GW9662 significantly inhibited alcohol-induced upregulation of MnSOD. Eight-week ethanol feeding significantly reduced cerebral I/R injury and alleviated the post-ischemic inflammatory response (upregulation of intercellular adhesion molecule-1 (ICAM-1) and E-selectin, microglial activation, and neutrophil infiltration). Treatment with GW9662 and endothelial-specific conditional knockout of PPARγ did not alter cerebral I/R injury and the inflammatory response in the control mice but abolish the neuroprotective effect in ethanol-fed mice. In addition, GW9662 and endothelial-specific conditional knockout of PPARγ diminished the inhibitory effect of LAC on the post-ischemic expression of adhesion molecules and neutrophil infiltration. Our findings suggest that LAC may protect against cerebral I/R injury by suppressing the post-ischemic inflammation via activation of PPARγ.

## 1. Introduction

One of the leading causes of permanent disability and mortality in adults is ischemic stroke, which occurs when an artery supplying blood to the brain gets obstructed [1,2]. Due to the use of tissue plasminogen activator (tPA) and endovascular thrombectomy for acute ischemic stroke, transient focal cerebral ischemia currently is a common type of ischemic stroke. Although recanalization and reperfusion therapies are very important for restoring blood flow to the hypoperfused area, reperfusion may paradoxically induce and worsen brain damage, named cerebral ischemia/reperfusion (I/R) injury [3]. The mechanisms underlying cerebral I/R injury include many interacting elements, such as oxidative/nitrosative stress, apoptosis, autophagy, and inflammation [3,4,5].

The post-ischemic inflammatory response is a double-edged sword. It produces secondary brain injury in the acute stage of stroke [6]. The initial post-ischemic inflammatory response is characterized by upregulation of adhesion molecules on brain microvascular endothelial cells, inflammatory activation of microglia, recruitment of leukocytes, and accumulation of cytokines and chemokines in the ischemic area. The activation of microglia and recruitment of leukocytes appear to be central features after transient focal cerebral ischemia [7,8]. Activated microglia directly contribute to brain I/R injury by phagocytosis and producing inflammatory and cytotoxic mediators [9]. On the other hand, when neutrophils were depleted from the circulation the infarct volume was reduced and cerebral blood flow was improved during the reperfusion [3,10]. The recruitment of leukocytes into the ischemic area is facilitated by the upregulation of adhesion molecules on endothelial cells prior to and during reperfusion [11]. In addition, the recruitment of leukocytes is associated with inflammatory activation of microglia and subsequent production of inflammatory mediators.

Alcohol is one of the most commonly used and abused chemical substances. It affects the incidence and prognosis of ischemic stroke [12,13,14,15,16,17,18,19]. Experimentally, we found that chronic alcohol consumption dose-dependently alters cerebral ischemia/reperfusion (I/R) injury in rodents [20,21,22]. Furthermore, the beneficial effect of low-dose alcohol consumption (LAC) may be related to an increased nuclear PPARγ expression/DNA-binding activation [23]. PPARγ is a ligand-activated transcription factor, which is localized in the cytoplasm and translocated to the nucleus upon ligand binding. It forms heterodimers with retinoid X receptors (RXRs) in the nucleus to regulate the transcription of various genes [24]. PPARγ is expressed in vascular endothelium and the central nervous system (CNS) [25,26]. Pharmacological activation of PPARγ has been shown to protect against cerebral I/R injury [27,28]. Although the precise mechanism remains to be defined, the neuroprotective effect of PPARγ activation appeared to relate to attenuation of post-ischemic inflammation [28]. Thus, our goal was to determine whether the beneficial effect of LAC against cerebral I/R injury is associated with PPARγ-mediated suppression of post-ischemic inflammation.

## 2. Results

### 2.1. Control Conditions

Eight weeks of daily feeding with 0.7 g/kg/day ethanol, two weeks of daily treatment with 3 mg/kg/day GW9662, and 3 weeks of daily treatment with 0.5 mg/kg/day tamoxifen did not significantly change body weight, mean arterial blood pressure (MABP), heart rate, and fasting glucose level when compared to the water group (Table 1).

### 2.2. Effect of Alcohol on PPARγ and MnSOD

**Figure 1:**
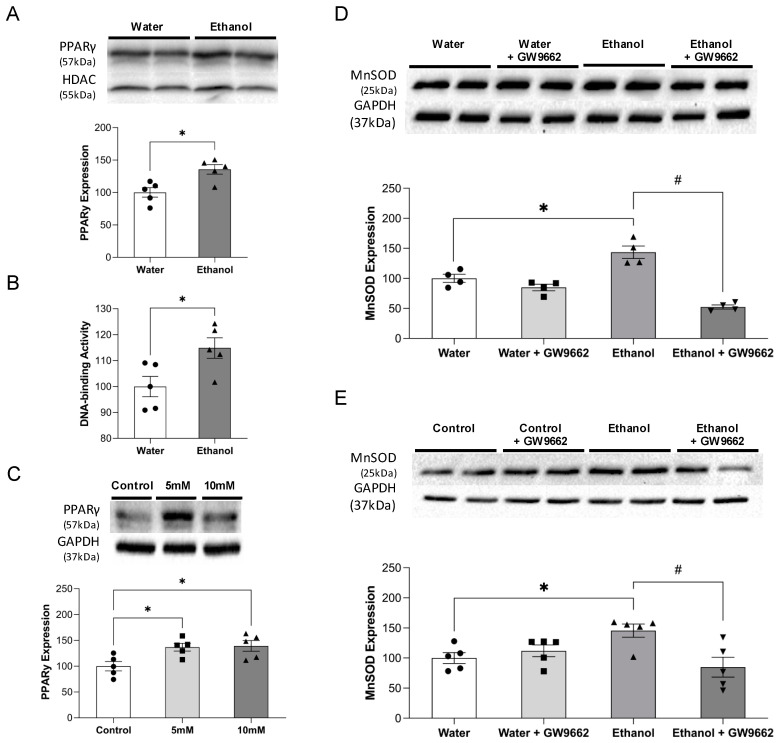
Effect of alcohol on nuclear protein expression and DNA-binding activity of PPARγ and protein expression of MnSOD. (**A**) Nuclear protein expression of PPARγ in the cerebral cortex of water and 0.7 g/kg/day ethanol-fed mice. Values are means ± SE. * *p* < 0.05. Analyzed using one-way ANOVA with Dunnett’s post hoc. (**B**) Nuclear DNA binding activity of PPARγ in the cerebral cortex of water and 0.7 g/kg/day ethanol-fed mice. Values are means ± SE. * *p* < 0.05. Analyzed using one-way ANOVA with Dunnett’s post hoc. (**C**) Protein expression of PPARγ in MBMVECs exposed to ethanol for 7 days. Values are means ± SE. * *p* < 0.05. Analyzed using one-way ANOVA with Dunnett’s post hoc. (**D**) Protein expression of MnSOD in the cerebral cortex of water and 0.7 g/kg/day ethanol-fed mice with or without GW9662 treatment. Values are means ± SE. * *p* < 0.05 vs. Water. ^#^ *p* < 0.05 vs. Ethanol. Analyzed using two-way ANOVA with Tukey’s post hoc. (**E**) Protein expression of MnSOD in 10 mM ethanol-exposed mice in the presence and absence of GW9662. Values are means ± SE. * *p* < 0.05 vs. Control. ^#^ *p* < 0.05 vs. Ethanol. Analyzed using two-way ANOVA with Tukey’s post hoc.

In in vivo study, 8-week consumption of 0.7 g/kg/day ethanol significantly increased nuclear PPARγ protein expression in the cerebral cortex (Figure 1A). Consistently, nuclear PPARγ DNA-binding activity of the cerebral cortex was significantly increased in the ethanol group compared to the water group (Figure 1B). In in vitro study, daily 2 h exposure with low-concentration ethanol for 7 days significantly upregulated PPARγ (Figure 1C). In addition to PPARγ, MnSOD was also upregulated in the cerebral cortex of ethanol-fed mice (Figure 1D). A similar increase was found in 7-day 10 mM ethanol-exposed MBMVEC (Figure 1E). Moreover, GW9662 (a selective PPARγ antagonist) abolished ethanol-induced upregulation of MnSOD both in vivo and in vitro (Figure 1D,E).

### 2.3. Effect of Chronic Alcohol Consumption on Cerebral I/R Injury

**Figure 2:**
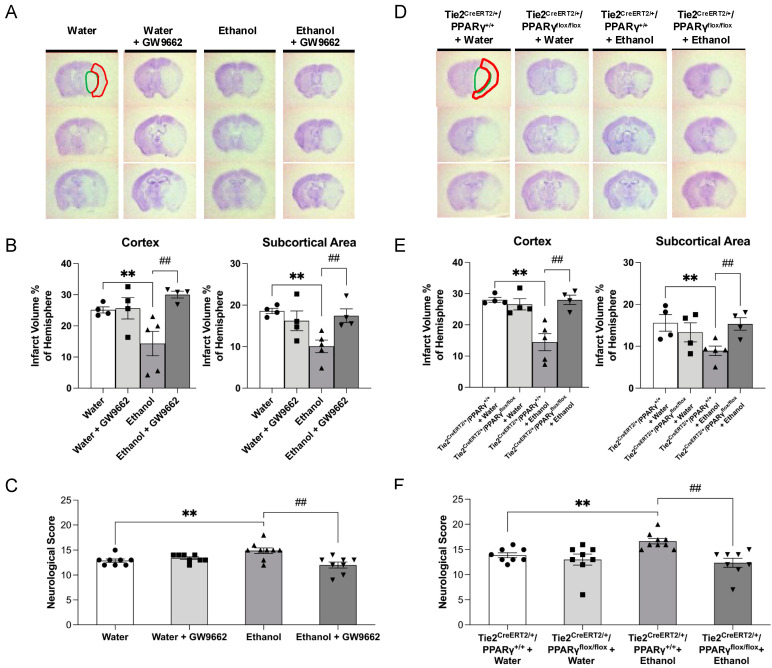
Effect of chronic ethanol consumption on cerebral I/R injury in the presence and absence of GW9662 and endothelial-specific conditional PPARγ knockout. (**A**,**D**) Representative brain sections stained with cresyl violet. (**B**,**E**) Infarct volume in the cerebral cortex and subcortical area. (**C**,**F**) Neurological deficit score. Values are means ± SE. ** *p* < 0.05 vs. Water or Tie2^CreERT2/+^/PPARγ^+/+^ + Water. ^##^ *p* < 0.05 vs. Ethanol or Tie2^CreERT2/+^/PPARγ^+/+^ + Ethanol. Analyzed using two-way ANOVA with Tukey’s post hoc.

Eight-week consumption of 0.7 g/kg/day ethanol before 90 min MCAO significantly reduced the infarct volume of the cerebral cortex and subcortical area and improved somatic sensorimotor function at 24 h of reperfusion in WT mice (Figure 2A–C). Two-week administration of PPARγ antagonist, GW9662, did not alter 90 min MCAO/24-h reperfusion-induced cerebral damage in the water-fed WT mice, but significantly alleviated the neuroprotective effect of ethanol (Figure 2A–C). On the other hand, the neuroprotective effect of ethanol was also found in Tie2^CreERT2/+^/PPARγ^+/+^ mice (Figure 2D–F). Three-week intraperitoneal injection of 0.5 mg/kg/day tamoxifen reduced PPARγ expression in the brain vascular endothelial cells of Tie2^CreERT2/+^/PPARγ^flox/flox^ mice, indicating the success of endothelial-specific PPARγ knockdown in the brain (see Supplemental Data). Endothelial-specific PPARγ knockdown did not further exacerbate 90 min MCAO/24-h reperfusion-induced cerebral damage in the water-fed Tie2^CreERT2/+^/PPARγ^flox/flox^ mice when compared to the water-fed Tie2^CreERT2/+^/PPARγ^+/+^ mice. However, endothelial-specific PPARγ knockdown significantly abolished the neuroprotective effect of ethanol on cerebral I/R damage (Figure 2D–F).

### 2.4. Effect of Chronic Alcohol Consumption on Protein Expression of Adhesion Molecules

**Figure 3:**
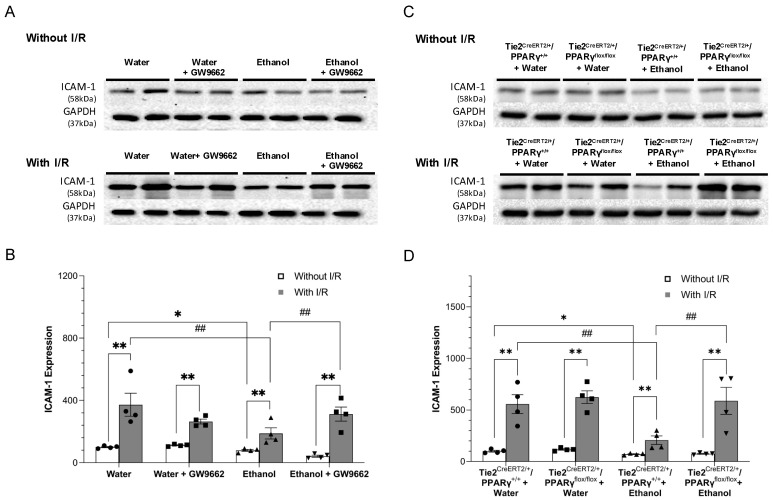
Effect of chronic ethanol consumption on ICAM-1 expression of the cerebral cortex in the presence and absence of GW9662 and endothelial-specific conditional PPARγ knockout. (**A**,**C**) Representative Western blots. (**B**,**D**) Values are means ± SE. * *p* < 0.05 vs. Water or Tie2^CreERT2/+^/PPARγ^+/+^ + Water. ** *p* < 0.05 vs. Without I/R. ^##^ *p* < 0.05 vs. Ethanol or Tie2^CreERT2/+^/PPARγ^+/+^ + Ethanol. Analyzed using two-way ANOVA with Tukey’s post hoc.

**Figure 4:**
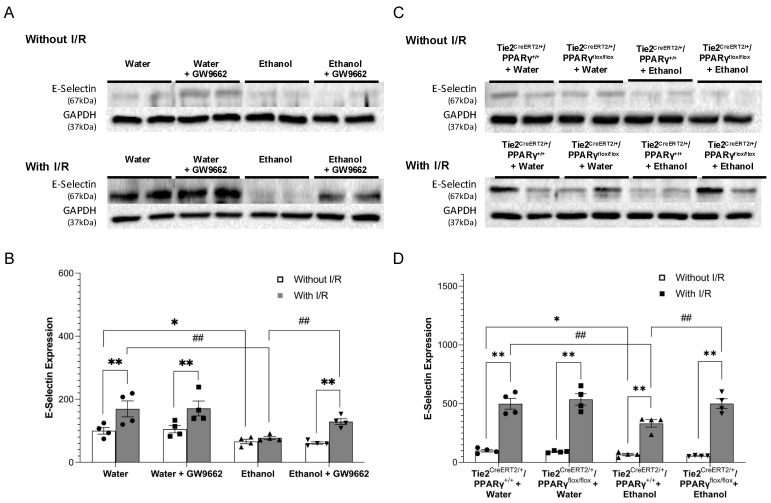
Effect of chronic ethanol consumption on E-selectin expression of the cerebral cortex in the presence and absence of GW9662 and endothelial-specific conditional PPARγ knockout. (**A**,**C**) Representative Western blots. (**B**,**D**) Values are means ± SE. * *p* < 0.05 vs. Water or Tie2^CreERT2/+^/PPARγ^+/+^ + Water. ** *p* < 0.05 vs. Without I/R. ^##^ *p* < 0.05 vs. Ethanol or Tie2^CreERT2/+^/PPARγ^+/+^ + Ethanol. Analyzed using two-way ANOVA with Tukey’s post hoc.

Eight-week consumption of 0.7 g/kg/day ethanol slightly but significantly downregulated baseline expression of ICAM-1 (Figure 3A,B) and E-selectin (Figure 4A,B) in WT mice. To avoid the potential influence of alcohol on total and/or regional cerebral blood flow, the peri-infarct cortex was selected as the area to compare the post-ischemic expression of ICAM-1 and E-selectin. Ninety-minute MCAO/24 h reperfusion significantly upregulated ICAM-1 and E-selectin in both water-fed and ethanol-fed WT mice. However, the magnitude of upregulation was significantly less in ethanol-fed WT mice (Figure 3A,B and Figure 4A,B). GW9662 administration did not alter baseline expression of both adhesion molecules in either water-fed or ethanol-fed WT-mice. In addition, GW9662 did not alter the 90 min MCAO/24-h reperfusion-induced upregulation of both adhesion molecules in water-fed WT mice. In contrast, GW9662 significantly suppressed the inhibitory effect of ethanol on the post-ischemic expression of ICAM-1 and E-selectin (Figure 3A,B and Figure 4A,B). On the other hand, a similar inhibitory effect of ethanol on the baseline and post-ischemic expression of ICAM-1 and E-selectin was observed in Tie2^CreERT2/+^/PPARγ^+/+^ mice (Figure 3C,D and Figure 4C,D). However, the inhibitory effect of ethanol on the post-ischemic expression of both ICAM-1 and E-selectin was diminished in Tie2^CreERT2/+^/PPARγ^flox/flox^ mice (Figure 3C,D and Figure 4C,D).

### 2.5. Effect of Chronic Alcohol Consumption on Neutrophil Infiltration

**Figure 5:**
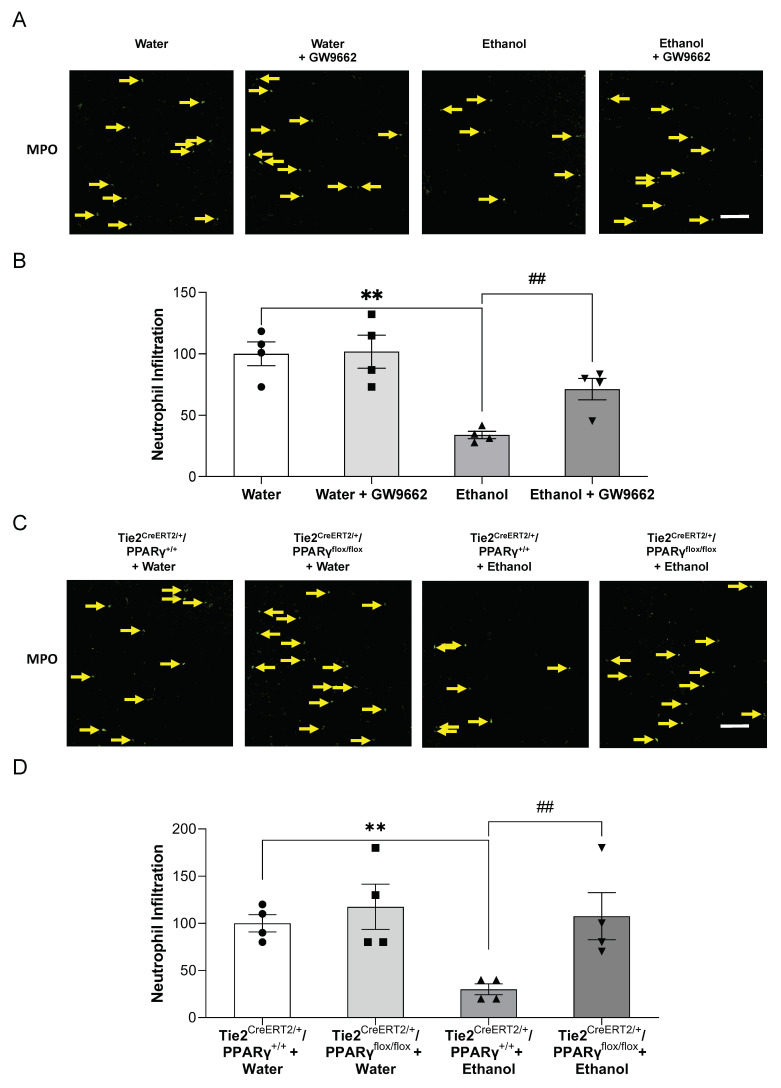
Effect of chronic ethanol consumption on neutrophil infiltration in the presence and absence of GW9662 and endothelial-specific conditional PPARγ knockout. (**A**,**C**) Representative immunohistochemistry staining of MPO (scale bar = 100 μm). (**B**,**D**) Values are means ± SE. ** *p* < 0.05 vs. Water or Tie2^CreERT2/+^/PPARγ^+/+^ + Water. ^##^ *p* < 0.05 vs. Ethanol or Tie2^CreERT2/+^/PPARγ^+/+^ + Ethanol. Analyzed using two-way ANOVA with Tukey’s post hoc.

Neutrophil infiltration was determined by immunofluorescence staining of MPO. Again, the peri-infarct cortex was chosen for avoiding the potential influence of alcohol on total and/or regional cerebral blood flow. Ninety-minute MCAO/24 h reperfusion produced a neutrophil infiltration in the ipsilateral hemisphere, but not the contralateral hemisphere of the MCAO. The neutrophil infiltration in the peri-infarct cortex was significantly decreased in ethanol-fed WT mice compared to water-fed WT mice (Figure 5A,B). GW9662 administration did not alter the neutrophil infiltration in the vehicle-fed WT mice, but significantly alleviated the inhibitory effect of ethanol on neutrophil infiltration (Figure 5A,B). Ninety-minute MCAO/24 h reperfusion-induced neutrophil infiltration in the peri-infarct cortex was also significantly inhibited in ethanol-fed Tie2^CreERT2/+^/PPARγ^+/+^ mice compared to vehicle-fed Tie2^CreERT2/+^/PPARγ^+/+^ mice (Figure 5C,D). The inhibitory effect was abolished by endothelial-specific PPARγ knockdown (Figure 5C,D).

### 2.6. Effect of Chronic Alcohol Consumption on Microglial Activation

**Figure 6:**
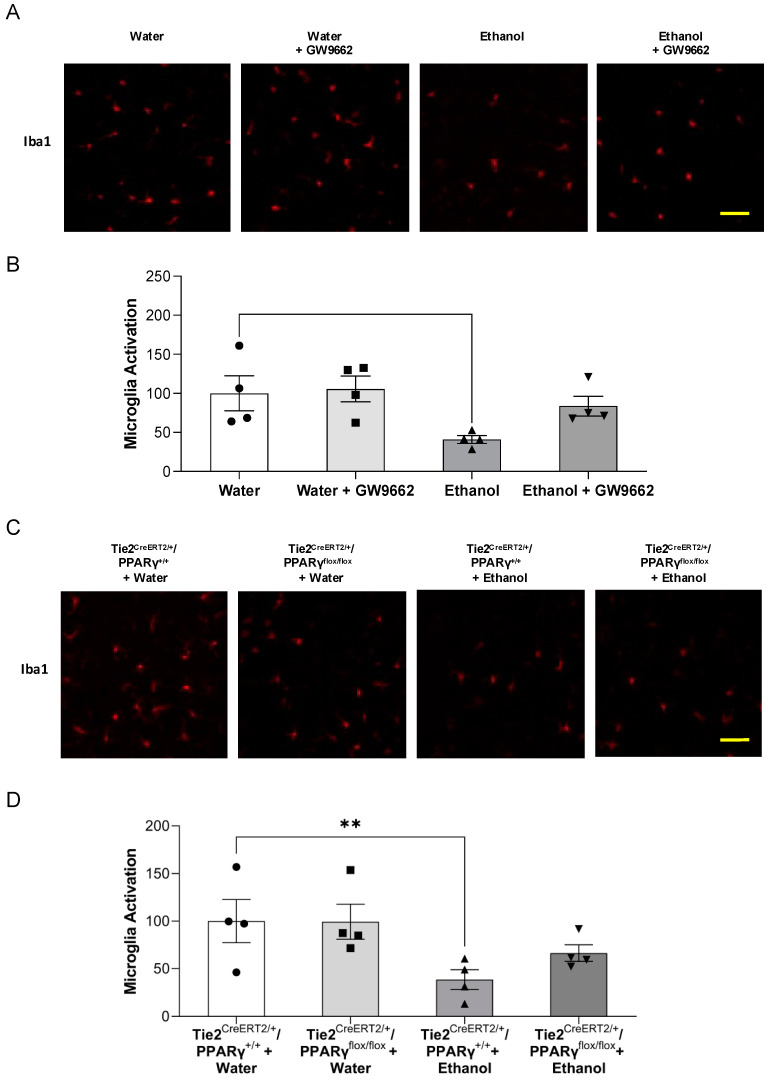
Effect of chronic ethanol consumption on microglia activation in the presence and absence of GW9662 and endothelial-specific conditional PPARγ knockout. (**A**,**C**) Representative immunohistochemistry staining of Iba1 (scale bar = 100 μm). (**B**,**D**) Values are means ± SE. ** *p* < 0.05 vs. Water or Tie2^CreERT2/+^/PPARγ^+/+^ + Water. Analyzed using two-way ANOVA with Tukey’s post hoc.

Microglial activation was assessed by immunofluorescence staining with Iba1. Ninety-minute MCAO/24-h reperfusion-induced microglial activation in the ipsilateral hemisphere of the MCAO. Eight-week consumption of 0.7 g/kg/day ethanol significantly inhibited post-ischemic microglial activation in the peri-infarct cortex of WT mice (Figure 6A,B). GW9662 administration did not alter the microglial activation in either water-fed WT mice or ethanol-fed WT mice (Figure 6A,B). On the other hand, the ethanol-induced inhibitory effect on post-ischemic microglial activation in the peri-infarct cortex was also observed in Tie2^CreERT2/+^/PPARγ^+/+^ mice. Endothelial-specific PPARγ knockdown did not alter the inhibitory effect of ethanol on post-ischemic microglial activation (Figure 6C,D).

## 3. Discussion

In the present study, the role of PPARγ in the inhibitory effect of LAC against the initial post-ischemic inflammatory response was investigated. There are five new findings. First, eight-week feeding with 0.7 g/kg/day significantly increased protein expression of MnSOD and nuclear protein expression/DNA-binding activity of PPARγ in the cerebral cortex. Second, chronic exposure of low-concentration ethanol upregulated MnSOD and PPARγ in MBMVECs. Third, ethanol-induced upregulation of MnSOD was diminished by PPARγ antagonist. Fourth, PPARγ antagonist and endothelial-specific PPARγ knockdown abolished the neuroprotective effect of LAC against cerebral I/R injury. Fifth, PPARγ antagonist and endothelial-specific PPARγ knockdown alleviated the inhibitory effect of ethanol on the post-ischemic expression of adhesion molecules and neutrophil infiltration. These findings complement and extend that which we have reported previously [21]. We speculate that LAC may protect against cerebral I/R injury by suppressing post-ischemic inflammation via activation of PPARγ.

Our recent studies found that 8-week gavage feeding with 0.7 g/kg/day ethanol prior to the ischemia significantly decreased infarct size and neurological deficits in a mouse model of transient focal cerebral ischemia [21,22]. The peak blood alcohol concentration was about 9 mM [21]. Blood alcohol level at 9 mM is commonly found after intake of one and a half American standard drinks (14 g of ethanol/each) [29,30]. Therefore, 5 mM and 10 mM were selected as low concentrations for ethanol in our in vitro study. Furthermore, blood alcohol concentration reduced rapidly and reached zero at 2 h after gavage feeding with 0.7 g/kg ethanol [21]. In in vitro study, we chose two-hour as daily exposure time for low-concentrations of ethanol.

A few studies have investigated the impact of alcohol on PPARγ expression/activity. Mitra et al. found that acute exposure with 100 mM ethanol downregulated PPARγ in human hepatoma cells [31]. Sun et al. reported that heavy ethanol consumption downregulated PPARγ in adipose tissue. Furthermore, Yeligar et al. reported that heavy ethanol consumption reduced PPARγ activity in alveolar macrophages [32]. Petersen found that PPARγ activity may be an important determinant of alcohol-related breast cancer [33]. In contrast, chronic heavy ethanol consumption upregulated PPARγ in the liver and pancreas [34,35,36]. In a previous study, we found that 8-week feeding with a liquid diet containing low-dose ethanol (5% of total calories) significantly increased nuclear expression and DNA-binding activity of PPARγ in the cerebral cortex [23]. Although the peak blood alcohol concentration was only about 1 mM, such peak was presumably reached several times a day since the liquid diet was the only source of food and water. Consistently, continuous exposure with 1 mM and 5 mM ethanol for 7 days upregulated PPARγ in CATH.a neuron [23]. In contrast, continuous exposure with 10 mM and 50 mM ethanol for 7 days downregulated PPARγ [23]. In the present study, gavage fed with 0.7 g/kg ethanol once a day for 8 weeks increased nuclear PPARγ expression and DNA-binding activity. In addition, daily 2 h exposure with 5 mM and 10 mM ethanol for 7 days significantly upregulated PPARγ. Although blood and medium alcohol concentrations in the present study were much higher than the ones in the previous study, the duration of exposure in the present study was much shorter than the one in the previous study. Thus, we speculate that the discrepancy regarding the effect of alcohol on PPARγ may be related to the dosage and duration of alcohol administration. In addition, it is also possible that the organ systems are differently affected by alcohol.

The post-ischemic activation of cerebral microvascular endothelial cells plays an important role in the pathogenesis of cerebral I/R injury by recruiting leukocytes and platelets to the ischemic region [37]. The activated endothelial cells upregulate adhesion molecules to promote the rolling and firm adhesion of both leukocytes and platelets. The recruitment of leukocytes and platelets leads to further injury by obstructing capillaries and releasing cytotoxic products. Several studies have found that strategies reducing and blocking ICAM-1, E-selectin, or P-selectin inhibited leukocyte infiltration, improved post-ischemic blood flow, and reduced cerebral I/R injury [38,39,40]. On the other hand, blocking VCAM-1 did not reduce leukocyte infiltration and cerebral I/R injury in either rats or mice [41]. In a recent study, we found that LAC significantly downregulated ICAM-1 and E-selectin either under basal conditions or at 24 h of reperfusion [21]. Thus, the inhibitory effect of LAC on ICAM-1 and E-selectin was further studied in the present study. PPARγ exerts an anti-inflammatory effect in vascular endothelial cells [25]. An early study reported that PPARγ agonists significantly suppressed tumor necrosis factor-induced expression of VCAM-1 and ICAM-1 in cultured human umbilical vein endothelial cells (HUVECs) and reduced monocyte/macrophage homing to atherosclerotic plaques in ApoE-deficient mice [42]. In addition, Wang et al. found that constitutive activation of PPARγ suppressed pro-inflammatory agonists-induced upregulation of ICAM-1, VCAM-1, and E-selectin in HUVECs [43]. Verrier et al. found that PPARγ agonists abolished high glucose-induced upregulation of ICAM-1, VCAM-1, and E-selectin in HUVECs [44]. Furthermore, PPARγ agonist significantly decreased the post-ischemic expression of ICAM-1, E-selectin, and P-selectin and neutrophils infiltration in rats [45]. The mechanism underlying the inhibitory effect of PPARγ on pro-inflammatory agonists-induced upregulation of molecules is not entirely clear but may be related to the suppression of the AP-1 and NF-κB pathways [43,46]. In the present study, PPARγ antagonist and endothelial-specific PPARγ knockdown abolished the inhibitory effect of LAC on the post-ischemic expression of ICAM-1 and E-selectin. Consistently, both approaches increased post-ischemic neutrophil infiltration in LAC mice. Thus, we speculate that the inhibitory effect of LAC on post-ischemic upregulation of ICAM-1 and E-selectin may be related to increased activation of PPARγ in the endothelial cells. Interestingly, either PPARγ antagonist or endothelial-specific PPARγ knockdown did not alter baseline expression of ICAM-1 and E-selectin in both LAC and control groups. This result is in agreement with previous findings [42,46,47]. The mechanism underlying the downregulated baseline ICAM-1 and E-selectin during LAC, however, remains to be determined.

Microglia are resident immune cells playing a key role in the immune response of the brain. Following ischemia, microglia are activated and morphologically changed from the shape with a small soma and long fine processes to the one with a large cell body and short thick processes. Activated microglia can be detected as early as 30 min after the onset of ischemia [48]. The precise mechanisms of ischemia-induced microglial activation are still not clear but may be related to increased reactive oxygen species (ROS), necrotic cells, and impaired tissues [49]. A few studies found that the magnitude of microglial activation is positively correlated to the severity of the injury [50,51]. In addition, activated microglia accelerate and expand cerebral I/R injury via phagocytosis and production of cytokines, matrix metalloproteinases (MMPs), and ROS in the early phase of ischemic stroke [52]. PPARγ is expressed in microglia. Early studies found that PPARγ agonists inhibited microglial activation [53]. However, no studies that we are aware of have determined the influence of PPARγ on post-ischemic microglial activation. In the present study, PPARγ antagonist and endothelial-specific PPARγ knockdown tended to attenuate the inhibitory effect of LAC on post-ischemic microglial activation. However, their effects failed to reach statistically significant. Thus, it is possible that the inhibitory effect of LAC on post-ischemic microglia activation is not related to PPARγ. In addition, the post-ischemic microglia activation may be not associated with the severity of the injury and neutrophil infiltration in LAC mice.

In the present study, we also found that MnSOD was upregulated in the cerebral cortex of LAC mice and 7-day low-concentration alcohol-exposed MBMVECs. Furthermore, the upregulated MnSOD may be related to an increased PPARγ activation. These results are in agreement with a previous study that MnSOD may be a target gene of PPARγ [54]. MnSOD is the major mitochondria-resident antioxidant enzyme that detoxifies superoxide anions produced by the mitochondrial respiratory chain. Mitochondrial oxidative stress plays a critical role in the pathogenesis of neuronal degeneration and many other pathologic conditions (such as atherosclerosis, aging, hypertension, obesity, and diabetes), which are identified as risk factors for ischemic stroke [55,56,57]. Mitochondria are the main source of cellular reactive oxygen species (ROS) during reperfusion [58,59]. Mitochondrial ROS appear to exacerbate ROS production by NAD(P)H oxidase and from mitochondrial (auto-stimulation) via a ROS-dependent ROS production mechanism in the vasculature [59,60]. Overproduced mitochondrial ROS may contribute to cerebral I/R injury by intensifying inflammation [61,62]. Furthermore, reduced ROS may be involved in PPARγ activation-mediated inhibition in ICAM-1 expression [63]. Thus, it is conceivable that PPARγ-mediated upregulation of MnSOD may also contribute to the anti-inflammatory effect of LAC.

In summary, the present study found that PPARγ may play a significant role in the beneficial effect of LAC against post-ischemic inflammation and cerebral I/R injury. Chronic alcohol consumption significantly alters the pathogenesis of ischemic stroke. Understanding how alcohol affects ischemic stroke will not only advance clinical management of ischemic stroke in alcohol users but also lead to novel strategies to treat ischemic stroke in non-alcohol users.

## 4. Materials and Methods

### 4.1. Mouse Brain Microvascular Endothelial Cells

C57/BL6J mouse brain microvascular endothelial cells (MBMVECs) (CellBiologics, IL) were maintained at 37 °C in a humidified atmosphere of 5% CO_2_ and propagated in a CellBiologics Complete Mouse Endothelial Cell Medium (that contains essential and non-essential amino acids, vitamins, organic and inorganic compounds, hormones, growth factors, and trace minerals), supplemented with endothelial cell growth supplement, antibiotics, and fetal bovine serum. To examine the chronic effect of ethanol on PPARγ and MnSOD, the MBMVECs at passages 4–8 were exposed to 5 or 10 mM ethanol for 2 h once a day for 7 days. Protein expression of PPARγ and MnSOD in the presence and absence of 10 μM GW9662 was determined by Western blot analysis. To avoid the acute effect of alcohol, the last exposure to ethanol was 24 h before collecting the cells.

### 4.2. Animal Models

All of the procedures and protocols (P-18-039) were approved by the Institutional Animal Care and Use Committee (IACUC) at the Louisiana State University Health Science Center (LSUHSC)-Shreveport and performed in accordance with the National Institutes of Health *Guide for the Care and Use Laboratory Animals* and the ARRIVE *(Animal Research: Reporting* in Vivo *Experiments)* guidelines. Male C57BL/6J wild type (20–25 g, 10–12 weeks), tamoxifen-induced endothelial PPARγ knockout (Tie2^CreERT2/+^/PPARγ^flox/flox^) (20–25 g, 10–12 weeks), and its littermate control (Tie2^CreERT2/+^/PPARγ^+/+^) (20–25 g, 10–12 weeks) mice on a C57/BL6J background were used. Tamoxifen-induced endothelial PPARγ knockout mice were generated by crossbreeding male Tie2^CreERT2/+^ mice (kindly provided by Dr. Manikandan Panchatcharam) with female PPARγ^flox/flox^ mice (Stock #: 004584, Jackson Labs, ME). Genotypes were determined by polymerase chain reaction (PCR) analysis.

### 4.3. Ethanol Preconditioning and Treatment

Wild-type (WT) mice (*n* = 50) were randomly divided into four groups: water (*n* = 13), 0.7 g/kg/d ethanol (*n* = 13), water + GW9662 (*n* = 12), and 0.7 g/kg/d ethanol + GW9662 (*n* = 12). Tie2^CreERT2/+^/PPARγ^flox/flox^ mice (*n* = 16) and Tie2^CreERT2/+^/PPARγ^+/+^ mice (*n* = 16) were randomly divided into four groups, Tie2^CreERT2/+^/PPARγ^flox/flox^ + water (*n* = 8), Tie2^CreERT2/+^/PPARγ^flox/flox^ + 0.7 g/kg/d ethanol (*n* = 8), Tie2^CreERT2/+^/PPARγ^+/+^ + water (*n* = 8), and Tie2^CreERT2/+^/PPARγ^+/+^ + 0.7 g/kg/d ethanol (*n* = 8). Ethanol groups were gavage fed with 10 mL/kg 7% ethanol once a day for eight weeks. The water groups were gavage fed with 10 mL/kg water. From the 7th week, GW9662 (3 mg/kg/day, IP) was given to GW9662-treated groups for two weeks. From the 6th week, all Tie2^CreERT2/+^/PPARγ^flox/flox^ and Tie2^CreERT2/+^/PPARγ^+/+^ mice were treated with tamoxifen (0.5 mg/kg/day, IP) for 3 weeks. At the end of 8 weeks of feeding, body weight, blood pressure, heart rate, and fasting blood glucose were measured similarly as described previously [21]. Blood pressure and heart rate were measured using a CODA mouse tail-cuff system (Kent Scientific, Torrington, CT, USA). Prior to the actual measurement, mice were trained for three consecutive days to acclimate to the restraint device and to also having the tail-cuff placed on them. Fasting blood glucose was measured by Bayer Breeze2 Blood Glucose Meter (Bayer HealthCare, Mishawaka, IN, USA). Prior to the measurement, mice were fasted for 12 h during the daytime.

### 4.4. Nuclear PPARγ DNA-Binding Activity

Ten wide-type mice from the vehicle and 0.7 g/kg/d ethanol groups were euthanized and exsanguinated at the end of 8 weeks of feeding. The brains were removed and cut into six 1.75 mm-thick coronal sections. Under the microscope, the cortical tissues were collected from the parietal and temporal lobes. The nuclear fraction from the cortical tissues was isolated using FOCUS SubCell Kit (G-Biosciences, St. Louis, MO, USA) following the manufacturer’s instructions. The protein concentration was measured using a BCA assay (Thermo Scientific, Plainville, MA, USA). Nuclear PPARγ protein expression was measured by Western blot analysis as described below. Nuclear PPARγ DNA-binding activity was determined by a PPARγ transcription factor assay kit (Cayman, Ann Arbor, MI, USA) following the manufacturer’s protocol. In brief, 120 µg nuclear proteins were incubated with a biotin-labeled DNA probe containing a PPAR-specific double-stranded consensus sequence and a single-stranded capture region. The samples were digested with a double-stranded DNA-specific nuclease and subsequently transferred to a 96-well plate coated with single-stranded DNA complementary to the capture region. A chemiluminescent alkaline phosphatase substrate was added, and the output signal was measured using FLUOstar Omega microplate reader (BMG LABTECH, Cary, NC, USA).

### 4.5. Transient Focal Cerebral Ischemia

Eight mice from each group were subjected to transient focal cerebral ischemia, which was induced by unilateral MCAO for 90 min as described previously [21]. To avoid a possible effect of acute ethanol, ethanol was not given on the day before the experiment. Prior to the procedure, mice were anesthetized with isoflurane (induction at 5% and maintenance at 1.5%) in a gas mixture containing 30%O_2_/70%N_2_ via a facemask. Body temperature was maintained with a temperature-controlled heating pad (Harvard Apparatus, March, Germany) during the experiment. A laser-Doppler flow probe (PERIMED, PF 5010 LDPM Unit, Järfälla, Sweden) was attached to the right side of the dorsal surface of the skull to monitor regional cerebral blood flow (rCBF). The right common and external carotid arteries were exposed and ligated. The right middle cerebral artery (MCA) was occluded by inserting a silicon rubber-coated monofilament (Doccol Corporation, Sharon, MA, USA) from the basal part of the right external carotid artery and advanced cranially into the right internal carotid artery to the point where the right middle cerebral artery branched off from the right internal artery. The success of the MCAO was indicated by an immediate drop in rCBF. After the occlusion of the right MCA for 90 min, reperfusion was achieved by withdrawing the suture and reopening the right common carotid artery. Animals were allowed to recover for 24 h. A 24-point scoring system was used to evaluate sensorimotor deficits as described previously [21]. Six tests (spontaneous activity, symmetry of movement, floor walking, beam walking, symmetry of forelimbs, and climbing wall of wire cage) for motor function and two tests (response to vibrissae touch and reaction to touch on either side of trunk) for sensory function were graded on a scale of 0 to 3 each. Neurological scores were assigned as follows: 0, complete deficit; 1, definite deficit with some function; 2, decreased response or mild deficit; 3, no evidence of deficit/symmetrical responses [30].

### 4.6. Brain Collection and Processing

The brains were collected and processed as described previously [21]. Four mice from each group were euthanized and exsanguinated at 24 h of reperfusion. The brains were removed and cut into six 1.75 mm-thick coronal sections. Under the microscope, the infarct core was identified as an opaque area, and the area bordering 2 mm the infarct core was considered as the peri-infarct area [64,65]. A researcher who was blinded to the experimental groups collected cortical tissues from the peri-infarct area and contralateral corresponding area. The rest four mice from each group were anesthetized and perfused transcardially with 1X phosphate-buffered saline (PBS), followed by 4% paraformaldehyde in 0.1 M PBS at 24 h of reperfusion. The brains were removed, fixed overnight in 4% paraformaldehyde in 0.1 M PBS, dehydrated in a graded series of sugar solutions over the course of 72 h, then embedded in O.C.T. compound (Fisher Scientific, Plainville, MA, USA) and quickly frozen for 5 min in liquid nitrogen. The frozen brains were then cut into 14 μm coronal sections and placed on frost-free slides.

### 4.7. Nissl Staining

To measure the infarct size, sections from eight levels (between 2.9 mm rostral and 4.9 mm caudal to bregma) were selected for Nissl staining as described previously [22]. The sections were incubated in 0.01% cresyl violet acetate (Sigma, St. Louis, MO, USA) solution for 14 min at 60 °C, rinsed in distilled water, dehydrated in ethanol, cleaned in xylene, and covered with xylene-a based mounting media (VWR). The sections were photographed under 1.0× magnification (Olympus, Tokyo, Japan) and evaluated using ImageJ. Complete lack of staining defined the infarct lesion. The infarct size was calculated by integration of area of infarct lesion with the distance between coronal levels and expressed as a percentage of total hemispheric volume.

### 4.8. Western Blot Analysis

Protein expression of MnSOD, ICAM-1, E-selectin, and nuclear PPARγ was measured as described previously [21]. The MBMVECs and cortical tissue samples were homogenized in ice-cold lysis buffer (150 mmol/L NaCl, 50 mmol/L Tris-HCl, 10 mmol/L EDTA, 0.1% Tween-20, 1% Triton, 0.1% mercaptoethanol, 0.1 mmol/L phenylmethyl sulfonyl fluoride, 5 μg/mL leupeptin, and 5 μg/mL aprotinin, pH 7.4). Lysates were then centrifuged at 12,000× *g* for 20 min at 4 °C, and the supernatants were collected. The protein concentration of the supernatants was determined by the Bradford protein assay (Bio-Rad, CA). SDS-PAGE was performed on a 10% gel on which 20 μg of total protein per well was loaded. After SDS-PAGE, the proteins were transferred onto a polyvinylidene difluoride membrane. Immunoblotting was performed using mouse anti-PPARγ (ab41928, Abcam, Cambridge, UK), goat anti-MnSOD (sc-18503, Santa Cruz Biotechnology), mouse anti-ICAM-1 (sc-8439, Santa Cruz Biotechnology, Dallas, TX, USA), and rabbit anti-E-selectin (ab18981, Abcam, Cambridge, UK) as primary antibodies and peroxidase-conjugated goat anti-mouse, donkey anti-goat, and mouse anti-rabbit IgG as the secondary antibody. The target proteins were then detected using enhanced chemiluminescence (ECL) detection (Genesee Scientific for ICAM-1, MnSOD, and nuclear PPARγ; Thermo Scientific for E-selectin), and the bands were analyzed using ChemiDoc^TM^ MP Imaging System (Bio-Rad, Hercules, CA, USA). To quantify, protein expression of PPARγ, MnSOD, ICAM-1, and E-selectin were normalized to histone deacetylase (HDAC) or GAPDH and expressed as percentage changes to the vehicle without I/R.

### 4.9. Immunohistochemistry Staining

Three sections (1.21 mm rostral and 0.23 mm and 1.31 mm caudal to bregma) from each mouse were washed with 1X PBS, blocked with 10% Bovine Serum Albumin (BSA) for at least 1 h, and then incubated overnight at 4 °C with 1:100 rabbit anti-myeloperoxidase (MPO) (Abcam, Cambridge, UK) for visualization of neutrophils or 1:100 rabbit anti-ionized calcium-binding adaptor molecule 1 (Iba1) (Wako Chemicals Inc., Cape Charles, VA, USA) for visualization of microglia as primary antibodies. The sections were then incubated with 1:200 AlexaFluor 555 donkey anti-rabbit (Santa Cruz Biotechnology, Dallas, TX, USA) for one hour at room temperature. Sections were mounted with DAPI mounting medium with Vector shield and visualized using a fluorescence microscope (Nikon Eclipse Ts2). Cells positive for MPO represented infiltrating neutrophils. For quantitative analysis, positive cells were counted in three separate areas per section surrounding the infarct area. To quantify microglia, cells positive for Iba1 were observed and counted. Resting microglia present with long processes extending from their cell body. Resting microglia present with long processes extending from their cell body. Upon activation, microglia change form from highly ramified to completely lacking processes. In addition, activated microglia show increased Iba1 expression [66,67]. Thus, microglia with increased Iba1 expression and three processes or less were deemed to be activated [66,68].

### 4.10. Statistical Analysis

All the quantitative data are presented as means ± SE. Prism 9 was used for statistical analyses. The difference in PPARγ was evaluated by one-way ANOVA followed by Dunnett’s test for multiple comparisons to the water/control group. The difference in MnSOD, infarct size, neurological score, ICAM-1, E-selectin, neutrophil infiltration, and microglial activation between groups were evaluated by two-way ANOVA followed by Tukey’s test. The difference in ICAM and E-selectin between without I/R and with I/R was evaluated by an unpaired t-test. A *p*-value of 0.05 or less was considered to be significant.

## Figures and Tables

**Table 1:** Physiological parameters.

	Body Weight (g)	MABP (mmHg)	Heart Rate (bpm)	Fasting Blood Glucose (mg/dL)
Water	27 ± 0.6	85 ± 3	656 ± 22	142 ± 7
Water + GW9662	28 ± 0.6	82 ± 3	680 ± 32	167 ± 12
Ethanol	28 ± 0.5	85 ± 3	663 ± 41	134 ± 9
Ethanol + GW9662	28 ± 0.55	86 ± 1	699 ± 39	162 ± 22
Tie2^CreERT2/+^/PPARγ^+/+^ + Water	27 ± 1.3	84 ± 2	605 ± 23	131 ± 18
Tie2^CreERT2/+^/PPARγ^flox/flox^ + Water	27 ± 1.1	87 ± 2	664 ± 26	147 ± 8
Tie2^CreERT2/+^/PPARγ^+/+^ + Ethanol	28 ± 1.6	84 ± 9	666 ± 24	124 ± 10
Tie2^CreERT2/+^/PPARγ^flox/flox^ + Ethanol	28 ± 1.0	81 ± 5	607 ± 27	139 ± 11

## Data Availability

Data is contained within the article or supplementary material.

## Supplementary Materials

The following are available online at https://www.mdpi.com/article/10.3390/ijms22105121/s1.
