# Supplementary material for: Chronic Low-Dose Alcohol Consumption Attenuates Post-Ischemic Inflammation via PPARγ in Mice
Source: Int J Mol Sci. 2021 May 12;22(10):5121. doi: 10.3390/ijms22105121 (PMC8150922; doi:10.3390/ijms22105121)
Supplement: Supplementary file 1 [file ijms-22-05121-s001.zip › ijms-1195075-supplementary.pdf]

Supplemental Data

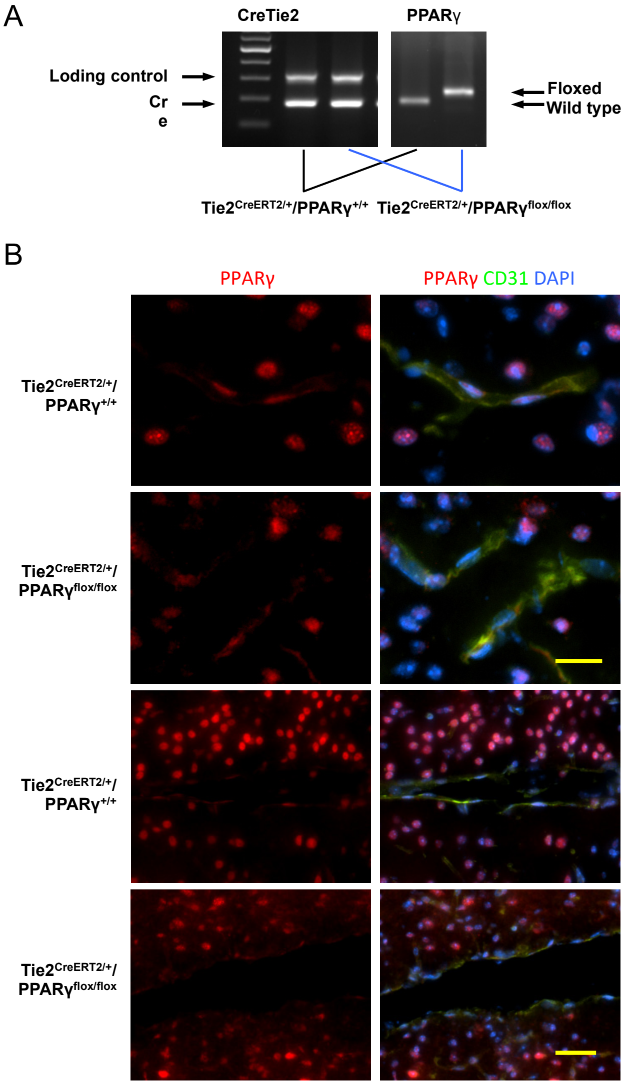

(**A**) PCR-based genotyping. The homozygous (fl/fl) mice with Cre positive were considered to be endothelial PPARγ knockout mice. (**B**) Double staining of PPARγ and CD31. Endothelial PPARγ expression was significantly reduced in both large and small brain vessels of Tie2$^{CreERT2/+}$/PPARγ$^{flox/flox}$ mice, indicating the success of endothelial specific PPARγ knockout in the brain.
